# Antioxidant and cytotoxic activities of *Dendrobium moniliforme* extracts and the detection of related compounds by GC-MS

**DOI:** 10.1186/s12906-018-2197-6

**Published:** 2018-04-23

**Authors:** Mukti Ram Paudel, Mukesh Babu Chand, Basant Pant, Bijaya Pant

**Affiliations:** 1 Central Department of Botany, Tribhuvan University, Kirtipur, Kathmandu, Nepal; 2 Annapurna Research Center, Kathmandu, Nepal

**Keywords:** *Dendrobium moniliforme*, DPPH, GC-MS, HeLa cell line, MTT, U251 cell line

## Abstract

**Background:**

The medicinal orchid *Dendrobium moniliforme* contains water-soluble polysaccharides, phenanthrenes, bibenzyl derivatives, and polyphenol compounds. This study explored the antioxidant and cytotoxic activities of *D. moniliforme* extracts and detected their bioactive compounds.

**Methods:**

Plant material was collected from the Daman of Makawanpur district in central Nepal. Plant extracts were prepared from stems using hexane, chloroform, acetone, ethanol and methanol. The total polyphenol content (TPC) in each extract was determined using Folin-Ciocalteu’s reagent and the total flavonoid content (TFC) in each extract was determined using the aluminium chloride method. The in vitro antioxidant and cytotoxic activities of each extract were determined using DPPH (2,2-diphenyl-1-picrylhydrazyl) and MTT (3-(4,5-dimethylthiazol-2-yl)-2,5-diphenyltetrazolium bromide) assays respectively. Gas chromatography and mass spectrometry (GC-MS) analysis was used to detect bioactive compounds.

**Results:**

TPC content was highest (116.65 μg GAE/mg of extract) in *D. moniliforme* chloroform extract (DMC) and TFC content was highest (116.67 μg QE/mg of extract) in *D. moniliforme* acetone extract (DMA). *D. moniliforme* hexane extract (DMH) extract showed the highest percentage of DPPH radical scavenging activity (94.48%), followed closely by *D. moniliforme* ethanol extract (DME) (94.45%), DMA (93.71%) and DMC (94.35%) at 800 μg/ml concentration. The antioxidant capacities of DMC, DMA, DMH and DME, which were measured in IC_50_ values, were much lower 42.39 μg/ml, 49.56 μg/ml, 52.68 μg/ml, and 58.77 μg/ml respectively than the IC_50_ of *D. moniliforme* methanol extract (DMM) (223.15 μg/ml). DMM at the concentration of 800 μg/ml most inhibited the growth of HeLa cells (78.68%) and DME at the same concentration most inhibited the growth of U251 cells (51.95%). The cytotoxic capacity (IC_50_) of DMM against HeLa cells was 155.80 μg/ml of extract and that of DME against the U251 cells was 772.50 μg/ml of extract. A number of bioactive compounds were detected in both DME and DMM.

**Conclusion:**

The fact that plant extract of *D. moniliforme* has a number of bioactive compounds which showed antioxidant and cytotoxic activities suggests the potential pharmacological importance of this plant.

**Electronic supplementary material:**

The online version of this article (10.1186/s12906-018-2197-6) contains supplementary material, which is available to authorized users.

## Background

*Dendrobium* is the second largest genus of the family Orchidaceae. There are 30 species in Nepal, distributed from tropical to temperate climatic regions [1]. It is an epiphytic orchid which is widely used in traditional medicine as a tonic and for treating human disorders [2–5]. One species of *Dendrobium*, *Dendrobium moniliforme* (synonym *D. candidum*), is a valuable medicinal orchid found across southeast and south Asia [1, 6, 7]. It contains water-soluble polysaccharides [8, 9], phenanthrenes, bibenzyl derivatives, and polyphenolic compounds [10–15].

Natural antioxidants and plant-derived compounds provide a strong defense against cellular damage caused by free-radical induced oxidative stress [16, 17]. Free-radicals and various reactive oxygen species are produced during cellular metabolism in all living systems and are responsible for oxidative cellular damage in human beings. To reduce this damage, some sort of defense mechanism is needed, and indeed, several types of natural and artificial antioxidants are used to control oxidative stress. In particular, plant-derived compounds are a potent source of novel antioxidant activity [18]. Bibenzyl derivatives isolated from *D. moniliforme* were examined for their antioxidant capacity using DPPH free-radical scavenging assay [11, 13, 14], a popular tool because of its simplicity and high sensitivity. This assay works on the principle that any hydrogen donor is an antioxidant. Thus, a compound’s antioxidant effect is proportional to the disappearance of DPPH radicals in test samples [19].

The natural antioxidant-rich compounds of *D. moniliforme* engage in many biological activities, including promoting the production of body fluids, serving neuroprotective, immunomodulatory and antioxidant functions [2, 20]. *D. candidum* has previously been shown to have in vitro anticancer effects on human carcinoma cells [21–27]. Indeed, it is often the case that bioactive agents of folk medicine help prevent the development of cancer by inducing apoptosis [28]. The induction of apoptosis in cancer cells is initially identified by morphological changes, including cell shrinkage, membrane blebbing, chromatin condensation, and nuclear fragmentation. The MTT assay can be used to screen the cytotoxicity of a crude extract [29]. More specifically, GC-MS can be used to detect and identify bioactive compounds in crude extracts [30, 31].

Despite the potential of *D. moniliforme*, little information exists on the antioxidant and cytotoxic activities of the crude extract of this orchid. To remedy that gap, the present study explored the antioxidant and cytotoxic activities of crude extracts of the plant and detected bioactive compounds present in it.

## Methods

### Plant material and the preparation of extract

The stems of *Dendrobium moniliforme* were collected from Daman of Makawanpur district in central Nepal. The plant was identified by Dr. Keshav Raj Rajbhandari. A voucher specimen of this plant is deposited in the Tribhuvan University Central Herbarium (TUCH) (voucher number – M02). The collected stems were air-dried and powdered. A Soxhlet extraction was used to prepare plant extracts with solvents of increasing polarity: hexane, chloroform, acetone, ethanol and methanol in the ratio of 1:10 (*w*/*v*) [32]. The solvents were then evaporated at room temperature to obtain dry extract and the extract was stored at 4 °C.

### Determination of the total polyphenol content in extract

The total polyphenol content (TPC) in each extract was determined using the Folin-Ciocalteu’s reagent method [33]. The reaction mixture was prepared by mixing 0.5 ml of a stock solution of extract, 2.5 ml of 10% Folin-Ciocalteu’s reagent, and 2.5 ml of 7.5% NaHCO_3_. In tandem, a blank solution was prepared from 0.5 ml of ethanol, 2.5 ml of 10% Folin-Ciocalteu’s reagent and 2.5 ml of 7.5% NaHCO_3_. The reaction mixtures were then incubated at room temperature for 45 min and their absorbance was measured using a Genesys UV-visible spectrophotometer at 765 nm. The TPC in extract was expressed as micrograms of gallic acid equivalent (GAE) per milligrams of dry extract.

### Determination of the total flavonoid content in extract

The total flavonoid content (TFC) in each extract was determined using the aluminium chloride method [33]. The reaction mixture was prepared by mixing 1 ml of a stock solution of extract and 1 ml of 2% AlCl_3_. In tandem, a blank solution was prepared by mixing 1 ml of ethanol and 1 ml of 2% AlCl_3_. The reaction mixtures were incubated for an hour at room temperature and then their absorbance was measured using a Genesys UV-visible spectrophotometer at 415 nm. The TFC in extract was expressed as micrograms of quercetin equivalent (QE) per milligrams of dry extract.

### Evaluation of the antioxidant activity of the extracts

The in vitro antioxidant activity of the extracts was determined using a DPPH (2,2-diphenyl-1-picrylhydrazyl) free-radical scavenging assay according to the method described in previous publication [34]. The stock solution of extract was diluted to prepare a series of concentrations (50, 100, 200, 400 and 800 μg/ml) for the antioxidant assay. An aliquot of 1.5 ml of 0.25 mM DPPH solution in ethanol and 1.5 ml of extract at each of the various concentrations was mixed. The mixture was shaken vigorously and allowed to sit for 30 min reach a steady state at room temperature. The decolourization of DPPH was determined by measuring the absorbance at 517 nm using a Genesys UV-visible spectrophotometer. The percentage of DPPH free-radical scavenging activity of each extract was then calculated using the following equation:

$$ \mathrm{Scavenging}\ \mathrm{rate}=\left[1-\left({\mathrm{A}}_1-{\mathrm{A}}_2\right)/{\mathrm{A}}_0\right]\times \kern0.37em 100\% $$

Where; A_0_ was the absorbance of the control (only DPPH, without extract), A_1_ was the absorbance of the extract with DPPH, and A_2_ was the absorbance of the extract without DPPH.

The antioxidant capacity of extract was expressed as the 50% inhibition of DPPH radicals (IC_50_ μg/ml of extract). The IC_50_ of extract was calculated using a polynomial regression equation in which the abscissa represents the series of concentrations of extract and the ordinate represents the mean of the triplicate percentage of antioxidant activity at each concentration.

### Evaluation of the cytotoxic activity of the extracts

The cytotoxic activity of the extracts was determined by using MTT assay [29, 35]. Human brain tumor cells (U251) and cervical cancer cells (HeLa) were cultured in RPMI 1640 medium and incubated under 5% CO_2_ at 37 °C for 48 h to reach 80% confluence. The cells were harvested by gently scraping them with a cell scraper and re-suspended in a medium. From the suspension, 5 × 10^3^ cells in 100 μl of medium were dispensed into each well of a 96-well microtiter cell culture plate and incubated under the same conditions for 48 h to allow time for the adherence and growth of cells. The supernatant was gently aspirated, and 100 μl of extracts were added with a range of four cytotoxic concentrations (100, 200, 400 and 800 μg/ml) prepared in a medium and incubated for 24 h. Ten microliter of 5 mg/ml MTT was added to every well and the plate was re-incubated for another 4 h. The formazan crystals formed were dissolved in 100 μl of DMSO. The plate was then read on a microplate reader (iMark™, Bio-Rad) at 595 nm. The number of dead cells per well was calculated as a percentage of the control, thereby measuring cell death after extract exposure. A dose-response curve was plotted for each extract to calculate the 50% inhibition of cell growth (IC_50_ μg/ml of extract). The cytotoxic capacities of extracts (IC_50_) were calculated using a regression equation in which the abscissa represents the series of concentrations of extract and the ordinate represents the mean of the triplicate percentage of inhibition of cell growth.

### Identification of the compounds in the extracts by GC-MS

GC-MS analysis of the bioactive compounds in the different extracts of the stems of *D. moniliforme* was conducted using GCMS-QP2010 Ultra (Shimadzu Europa GmbH, Germany) equipped with high-speed data acquisition and processing via advanced scan speed protocol. Spectroscopic detection by GC-MS involved an electron ionization system which utilized high-energy electrons (70 eV). Pure helium gas (99.99%) was used as the carrier gas with a column-flow rate of 0.95 ml/min. The initial temperature was set at 100 °C and increased at the rate of 3 °C/min after a holding time of about 10 min. In the end, the temperature was increased to 300 °C at 10 °C/min. One microliter of 1% extract diluted with respective solvents was injected in a splitless mode. The relative quantity of the chemical compounds present in each of the extract was expressed as a percentage based on the peak area produced in the chromatogram. Bioactive compounds were identified based using GC retention times and by matching the spectra with standard values using computer software.

### Statistical analysis

All data are expressed as a mean of three analyses. A linear regression model was applied to obtain equations for standards, gallic acid and quercetin. The total polyphenol and flavonoid contents were calculated by using linear equation of standards. The Duncan multiple-range test was applied to compare the significance differences of the total polyphenol and flavonoid contents in the extracts at *p* ≤ 0.05. The IC_50_ values of the antioxidant and cytotoxic activities of the extracts were calculated by using an appropriate linear or non-linear regression equation obtained from the percentage activity of each extract at various concentrations with the F-statistic at *p* ≤ 0.05. All the analysis was done using statistical software R version 3.1.2 [36].

## Results

### Plant extracts have significant amounts of total polyphenol and flavonoid contents

**Fig. 1:**
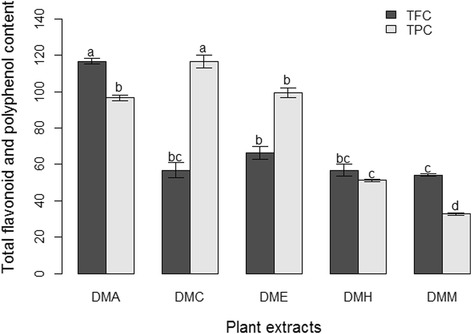
Total polyphenol and flavonoid contents in crude extracts of *D. moniliforme* (*n* = 3; *p* ≤ 0.05; values of the same letters are not statistically different)

Total polyphenol contents (TPCs) in the extracts were calculated using the linear equation for standard gallic acid (y = 0.0154 x – 0.3285; *R*^*2*^ = 0.989; *p* = 0.005) and total flavonoid contents (TFCs) was calculated using the linear equation for standard quercetin hydrate (y = 0.0242 x – 0.1845; *R*^*2*^ = 0.976; *p* = 0.012). The TPCs in the extracts ranged from 32.68 to 116.65 μg GAE/mg of extract and TFCs from 54.13 to 116.67 μg QE/mg of extract (Additional file 1). The TPC of DMC (116.65 μg GAE/mg of extract) was much greater than those of other extracts. The TPCs of DME and DMA were the next highest respectively. The TFC of DMA (116.67 μg QE/mg of extract) was much higher than it was in other extracts, though the TFC of DME was also high (Fig. 1). The values of TPC and TFC in all the extracts were statistically significantly different at *p* ≤ 0.05.

### Plant extracts showed the significant antioxidant activity by reducing DPPH radical

**Fig. 2:**
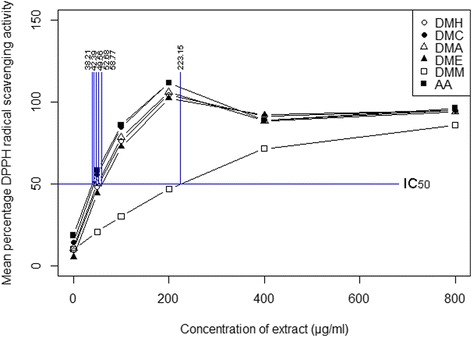
Percentage of DPPH free-radical scavenging activity of crude extracts of *D. moniliforme* and ascorbic acid and their antioxidant capacities (*n* = 3; *p* ≤ 0.05)

The antioxidant activity of different extracts was determined by DPPH free-radical scavenging assay. The mean percentage of DPPH free-radical scavenging activity at different concentrations of extracts is shown in Fig. 2. DMH had the highest percentage of DPPH free-radical scavenging activity (94.48%), followed by DME (94.45%), DMC (94.35%), and DMA (93.71%), and, considerably lower by DMM (86.92%) at their 800 μg/ml concentration. The percentage of DPPH free-radical scavenging activity was measured at a range of concentrations from 50 to 800 μg/ml (Additional file 2). In all cases, it increased as the concentration increased. The antioxidant capacity of each extract was measured as the IC_50_ value of the extract, or in other words, the amount required to scavenge 50% DPPH free-radicals.The antioxidant capacities of DMC, DMA, DMH and DME had much lower IC_50_ values – 42.39 μg/ml, 49.56 μg/ml, 52.68 μg/ml and 58.77 μg/ml respectively than did DMM (223.15 μg/ml). Except for DMM, the IC_50_ values of other extracts were statistically similar to the ascorbic acid (AA), whose IC_50_ value was 38.21 μg/ml (Fig. 2).

### Plant extracts showed the significant cytotoxic activity against the HeLa and U251 cell lines

**Fig. 3:**
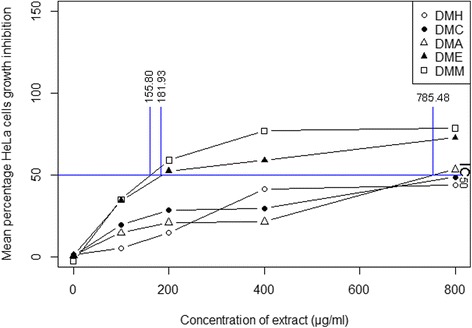
Percentage of cell growth inhibition of the HeLa cell line by crude extracts of *D. moniliforme* and their cytotoxic capacities (*n* = 3; *p* ≤ 0.05)

**Fig. 4:**
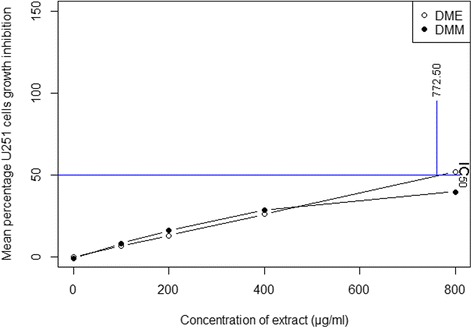
Percentage of cell growth inhibition of the U251 cell line by crude extracts of *D. moniliforme* and their cytotoxic capacities (*n* = 3; *p* ≤ 0.05)

The cytotoxic activity of extracts of *D. moniliforme* against the HeLa and U251 cell lines was determined using MTT colorometric assay. The mean percentage of the cytotoxic activity of extracts of different concentrations is shown in Fig. 3 (for HeLa cells) and in Fig. 4 (for U251 cells). The percentage of cell growth inhibition of extract-treated cells is increased as the concentration of the extract increased (Additional file 3 for HeLa cells and Additional file 4 for U251 cells). DMM at the concentration of 800 μg/ml showed the highest inhibition percentage of HeLa cells growth (78.68%), while DME at the concentration of 800 μg/ml had the highest cell growth inhibition of U251 cells (51.95%). DME at 800 μg/ml showed moderate inhibition of the growth of HeLa cells (72.66%) and DMM at 800 μg/ml showed moderate inhibition of the growth of U251 cells (39.93%). The cytotoxic capacity of an extract was measured as its IC_50_ value, or the amount of extract required to inhibit the 50% cell growth. The cytotoxic capacities of DMM and DME against HeLa cells were 155.80 μg/ml and 181.93 μg/ml of extract respectively (Fig. 3). Their cytotoxic capacities were statistically significantly different from those of other extracts. In terms of inhibiting the growth of U251 cells, the cytotoxic capacity of DME was 772.50 μg/ml of extract, but neither DMH, DMC nor DMA showed any such capacity (Fig. 4).

### Plant extracts have phenol derivatives detected by GC-MS

The bioactive compounds present in the hexane, chloroform, acetone, ethanol and methanol extracts obtained from *D. moniliforme* stems were detected using gas chromatography (GC) and identified through mass spectrometry (MS). The compounds detected and identified in DME and DMM are shown in Tables 1 and 2. The results of the GC-MS analysis of the other extracts are not shown here because those extracts showed either no or little cytotoxic activity. The elution time of compounds with their base mass-to-charge ratio (m/z) and their contents by percentage were also determined. Based on abundance, two major compounds dimethylsulfoxonium formylmethylide (37.99%) and 2,3-dihydro-3,5-dihydroxy-6-methyl-4H-pyran-4-one (21.41%) were detected and identified in DMM and two, 5-(hydroxymethyl)-2-furancarboxaldehyde (45.59%) and 2,3-dihydro-3,5-dihydroxy-6-methyl-4H-pyran-4-one (16.74%) in DME. Both DME and DMM had a number of other phenol derivatives too including tetrahydro-1,1-dioxide-thiophene-3-ol, γ-sitosterol, 2-methoxy-4-vinylphenol, 2,6-dimethoxy-phenol, 2,6-dimethoxy-4-(2-propenyl)-phenol, n-nonadecanol-1, 1-heptacosanol and stigmast-5-en-3-ol. The GC chromatogram of DMM and DME are presented in Additional files 5 and 6 respectively.

**Table 1:** Bioactive compounds detected in *D. moniliforme* ethanol extract (DME)

S.N.	Compound Name	RT (min)	Content (%)	Base m/z
1	Dimethylsulfoxonium formylmethylide	4.043	4.50	63.00
2	4H-Pyran-4-one, 2,3-dihydro-3,5-dihydroxy-6-methyl-	4.142	16.74	43.00
3	3-Cyclohexene-1-methanol,.alpha.,alpha.,4-trimethyl-,(S)-	4.307	2.51	59.05
4	3(2H)-Furanone, 4-methoxy-2,5-dimethyl-	4.367	3.95	43.00
5	Thiophene-3-ol, tetrahydro-, 1,1-dioxide	4.410	3.17	44.00
6	5-Acetoxymethyl-2-furaldehyde	4.480	4.52	126.10
7	2-Furancarboxaldehyde, 5-(hydroxymethyl)-	4.754	45.59	97.05
8	5-Acetoxymethyl-2-furaldehyde	5.010	2.66	126.10
9	2-Methoxy-4-vinylphenol	5.131	2.61	150.20
10	Phenol, 2,6-dimethoxy-	5.371	2.70	154.20
11	4-Methyl-2,5-dimethoxybenzaldehyde	6.991	2.75	180.15
12	Cinnamic acid, 4-hydroxy-3-methoxy-,	7.103	0.96	149.15
13	Phenol, 2,6-dimethoxy-4-(2-propenyl)-	7.994	0.83	194.15
14	4-((1E)-3-Hydroxy-1-propenyl)-2-methoxyphenol	8.414	1.19	137.15
15	Pentadecanoic acid	9.688	0.76	43.05
16	Methyl (3,4-dimethoxyphenyl)(hydroxy) acetate	9.970	1.01	167.15
17	Benzenemethanol, 2,5-dimethoxy-, acetate	10.128	0.96	210.15
18	Bis[(4-methoxyphenyl)methyl]disulfide	12.852	1.34	121.15
19	Tetradecanal	13.308	0.67	57.10
20	.gamma.-Sitosterol	21.005	0.60	43.05

**Table 2:** Bioactive compounds detected in *D. moniliforme* methanol extract (DMM)

S.N.	Compound Name	RT (min)	Content (%)	Base m/z
1	Dimethylsulfoxonium formylmethylide	4.081	37.99	63.00
2	Methoxyacetic acid, 2-pentyl ester	4.217	7.74	43.00
3	4H-Pyran-4-one, 2,3-dihydro-3,5-dihydroxy-6-methyl-	4.303	21.41	43.00
4	3-Cyclohexene-1-methanol,.alpha.,alpha.,4-trimethyl-,(S)-	4.481	9.62	61.00
5	Dimethylsulfoxonium formylmethylide	4.557	3.01	78.00
6	2-Furancarboxaldehyde, 5-(hydroxymethyl)-	4.686	6.41	97.05
7	2-Methoxy-4-vinylphenol	5.199	0.39	150.15
8	Phenol, 2,6-dimethoxy-	5.411	0.58	154.15
9	3′,5′-Dimethoxyacetophenone	6.961	0.90	180.15
10	Diethyl Phthalate	7.089	0.29	149.10
11	Phenol, 2,6-dimethoxy-4-(2-propenyl)-	7.980	0.31	194.10
12	1,2-Benzenedicarboxylic acid, bis(2-methylpropyl) ester	9.064	7.11	149.10
13	Octatriacontyl pentafluoropropionate	13.036	0.74	57.05
14	Heneicosane	14.459	0.29	57.10
15	n-Nonadecanol-1	14.510	0.70	83.10
16	Cyclononasiloxane, octadecamethyl-	15.101	0.19	73.05
17	1-Heptacosanol	15.957	0.65	57.10
18	1-Heptacosanol	17.667	0.49	97.10
19	Stigmast-5-en-3-ol, oleate	18.154	0.31	97.10
20	.gamma.-Sitosterol	20.976	0.85	43.05

## Discussion

The results showed that variations in the TPC and TFC of different extracts are affected by the polarity of the solvent used and the nature of the phytochemicals dissolved. The fact that extraction solvents with different polarities extract varying amounts of polyphenol and flavonoid compounds from plants is well known [37–40]. This study used a range of extraction solvents, from those with low polarity to those with high polarity, in order to dissolve phytochemicals with similar polarities. The hexane extract (DMH) obtained from using hexane, a low-polarity solvent, had little TPC or TFC because it has very few phytochemicals derived from either polyphenol or flavonoid groups. In contrast, both chloroform and acetone extracts exhibited high TPC and TFC, probably because these extracts contain many compounds of polyphenol and flavonoid groups with polarities similar to those of chloroform and acetone. Previous research on the extraction of differing flavonoid and polyphenol contents and compounds in different solvents in various *Dendrobium* species supports the present research [37, 41–44]. The types of compounds we identified in *D. moniliforme* in this study were similar to those identified in this plant in previous studies [10–15].

The antioxidant potential of each extract was measured using the change in its absorbance of decolourized DPPH free-radicals as it accepts electrons from the antioxidant-rich compounds. Previous research found that phenol derivatives have the ability to reduce and decolourize DPPH free-radicals [45–47]. The fact that the antioxidant activity of the extracts has a strong relationship with the solvent employed is mainly due to the fact that compounds with antioxidant potential dissolve differentially in solvents with different polarities [37, 48]. Natural antioxidants present in plants inhibit the deleterious consequences of oxidative stress. In particular, the literature reports that the polyphenol and flavonoid contents of extracts of the *Dendrobium* species exhibit antioxidant activity [41, 44, 49–51]. For example, bibenzyl derivatives isolated from *D. moniliforme* showed antioxidant activity [11, 13, 14]. This study found that the antioxidant potential of four extracts of *D. moniliforme* is due to the presence of antioxidant-rich compounds like phenol derivatives.

Free-radicals and reactive oxygen species induce the carcinogenesis of human cells that causes cancer [16]. Antioxidant-rich compounds in food products, in contrast, scavenge free-radicals and prevent the radical chain reaction of oxidation, thereby delaying or inhibiting the oxidation process. The antioxidant effect of phenol derivatives accounts for their cancer chemo-preventive and therapeutic effects [52]. The mechanisms which phenol derivatives use to inhibit cancer, arresting the cell cycle and inducing apoptosis is described in study of the anticancer activity of *D. moniliforme* [53, 54]. This study found that both methanol and ethanol extracts were cytotoxic against the HeLa and U251 cell lines. These both extracts have many phenol derivatives capable of reducing the number of free radicals and inhibiting oxidation. The several phenol derivatives detected in these extracts arrest the cell cycle and induce apoptosis through morphological changes like cell shrinkage, chromatin condensation, and nuclear fragmentation [28, 55, 56], thereby serving as an important defense mechanism preventing the proliferation of cancer cells. Like other studies of the anticancer activities of *Dendrobium* species [21–27], this study too, found that phenol derivatives induces the apoptosis of cancer cells. Phenol derivatives of extracts do exhibit both antioxidant and anticancer effects [2–4, 57].

## Conclusions

*D. moniliforme* extracts contain a number of bioactive compounds which exhibit both antioxidant and cytotoxic activities against free radicals and cancer cell lines respectively. This result suggests that this plant has potential pharmacological importance.

## Additional files

Additional file 1: Total polyphenol or flavonoid contents in the plant extracts of *D. moniliforme* (triplicate data). (DOCX 12 kb)

Additional file 2: Percentage of DPPH free-radical scavenging activity by plant extracts of *D. moniliforme* (triplicate data). (DOCX 12 kb)

Additional file 3: Percentage of HeLa cell growth inhibition by plant extracts of *D. moniliforme* (triplicate data). (DOCX 12 kb)

Additional file 4: Percentage of U251 cell growth inhibition by plant extracts of *D. moniliforme* (triplicate data). (DOCX 11 kb)

Additional file 5: Chromatogram of *D. moniliforme* methanol extract (DMM). (DOCX 142 kb)

Additional file 6: Chromatogram of *D. moniliforme* ethanol extract (DME). (DOCX 143 kb)

## Abbreviations

AA: Ascorbic acid
DMA: *Dendrobium moniliforme* acetone extract
DMC: *Dendrobium moniliforme* choloroform extract
DME: *Dendrobium moniliforme* ethanol extract
DMH: *Dendrobium moniliforme* hexane extract
DMM: *Dendrobium moniliforme* methanol extract
DPPH: 2,2-diphenyl-1-picrylhydrazyl
MTT: 3-(4,5-dimethylthiazol-2-yl)-2,5-diphenyltetrazolium bromide
TFC: Total flavonoid content
TPC: Total polyphenol content
