# Supplementary material for: Antioxidant and cytotoxic activities of Dendrobium moniliforme extracts and the detection of related compounds by GC-MS
Source: BMC Complement Altern Med. 2018 Apr 23;18:134. doi: 10.1186/s12906-018-2197-6 (PMC5913799; doi:10.1186/s12906-018-2197-6)
Supplement: Supplementary file 1 — Total polyphenol or flavonoid contents in the plant extracts of D. moniliforme (triplicate data). (DOCX 12 kb) [file 12906_2018_2197_MOESM1_ESM.docx]

| **Plant Extracts** | **Total polyphenol or flavonoid content** | **Amount in μg/mg extract** |
| --- | --- | --- |
| DMH | TPC | 52.63 |
| DMH | TPC | 50.49 |
| DMH | TPC | 50.94 |
| DMC | TPC | 123.99 |
| DMC | TPC | 113.15 |
| DMC | TPC | 112.82 |
| DMA | TPC | 99.38 |
| DMA | TPC | 96.53 |
| DMA | TPC | 93.99 |
| DME | TPC | 95.23 |
| DME | TPC | 99.06 |
| DME | TPC | 104.45 |
| DMM | TPC | 33.80 |
| DMM | TPC | 31.92 |
| DMM | TPC | 32.31 |
| DMH | TFC | 57.93 |
| DMH | TFC | 61.74 |
| DMH | TFC | 50.83 |
| DMC | TFC | 51.98 |
| DMC | TFC | 53.64 |
| DMC | TFC | 65.04 |
| DMA | TFC | 119.75 |
| DMA | TFC | 115.29 |
| DMA | TFC | 114.96 |
| DME | TFC | 73.31 |
| DME | TFC | 62.40 |
| DME | TFC | 63.22 |
| DMM | TFC | 54.63 |
| DMM | TFC | 52.81 |
| DMM | TFC | 54.96 |

**Additional file 1**

Total polyphenol or flavonoid contents in the plant extracts of *D. moniliforme* (triplicate data)
