# Supplementary material for: Antioxidant and cytotoxic activities of Dendrobium moniliforme extracts and the detection of related compounds by GC-MS
Source: BMC Complement Altern Med. 2018 Apr 23;18:134. doi: 10.1186/s12906-018-2197-6 (PMC5913799; doi:10.1186/s12906-018-2197-6)
Supplement: Supplementary file 2 — Percentage of DPPH free-radical scavenging activity by plant extracts of D. moniliforme (triplicate data). (DOCX 12 kb) [file 12906_2018_2197_MOESM2_ESM.docx]

**Additional file 2**

Percentage of DPPH free-radical scavenging activity by plant extracts of *D. moniliforme* (triplicate data).

| **Concentration of extract in μg/ml** | **DMH** | **DMC** | **DMA** | **DME** | **DMM** |
| --- | --- | --- | --- | --- | --- |
| 800 | 94.29 | 94.68 | 94.09 | 94.87 | 87.61 |
| 800 | 93.61 | 94.48 | 93.90 | 93.90 | 84.07 |
| 800 | 95.55 | 93.90 | 93.13 | 94.58 | 89.09 |
| 400 | 94.97 | 94.77 | 92.26 | 94.09 | 73.55 |
| 400 | 93.71 | 93.03 | 92.64 | 94.48 | 73.45 |
| 400 | 94.00 | 94.29 | 92.45 | 94.29 | 53.79 |
| 200 | 94.09 | 94.68 | 92.26 | 94.29 | 56.44 |
| 200 | 94.19 | 94.48 | 92.35 | 94.09 | 38.25 |
| 200 | 94.19 | 92.84 | 92.55 | 94.29 | 51.72 |
| 100 | 85.96 | 92.64 | 91.29 | 90.42 | 34.12 |
| 100 | 69.70 | 94.48 | 81.32 | 62.15 | 32.35 |
| 100 | 81.03 | 93.42 | 81.12 | 81.41 | 27.73 |
| 50 | 60.21 | 73.09 | 75.70 | 45.30 | 30.38 |
| 50 | 63.70 | 82.96 | 62.83 | 50.44 | 31.17 |
| 50 | 63.21 | 64.67 | 55.76 | 54.50 | 32.35 |
