# Supplementary material for: Antioxidant and cytotoxic activities of Dendrobium moniliforme extracts and the detection of related compounds by GC-MS
Source: BMC Complement Altern Med. 2018 Apr 23;18:134. doi: 10.1186/s12906-018-2197-6 (PMC5913799; doi:10.1186/s12906-018-2197-6)
Supplement: Supplementary file 3 — Percentage of HeLa cell growth inhibition by plant extracts of D. moniliforme (triplicate data). (DOCX 12 kb) [file 12906_2018_2197_MOESM3_ESM.docx]

**Additional file 3**

Percentage of HeLa cell growth inhibition by plant extracts of *D. moniliforme* (triplicate data).

| **Concentration of extract in μg/ml** | **DMH** | **DMC** | **DMA** | **DME** | **DMM** |
| --- | --- | --- | --- | --- | --- |
| 800 | 46.56 | 52.46 | 51.80 | 70.16 | 76.07 |
| 800 | 40.65 | 44.84 | 54.84 | 75.16 | 81.29 |
| 800 | 42.35 | 46.45 | 52.56 | 72.07 | 78.61 |
| 400 | 41.25 | 32.19 | 20.00 | 60.00 | 75.31 |
| 400 | 42.90 | 28.09 | 24.07 | 58.95 | 74.69 |
| 400 | 42.25 | 28.95 | 22.00 | 59.95 | 74.00 |
| 200 | 11.93 | 26.61 | 18.96 | 53.82 | 69.42 |
| 200 | 10.76 | 25.00 | 18.67 | 45.57 | 63.61 |
| 200 | 10.25 | 25.67 | 18.00 | 48.57 | 65.07 |
| 100 | 9.20 | 21.47 | 17.79 | 36.50 | 26.07 |
| 100 | 9.54 | 23.38 | 16.31 | 38.15 | 27.08 |
| 100 | 9.25 | 21.84 | 16.33 | 37.95 | 27.00 |
