# Supplementary material for: Antioxidant and cytotoxic activities of Dendrobium moniliforme extracts and the detection of related compounds by GC-MS
Source: BMC Complement Altern Med. 2018 Apr 23;18:134. doi: 10.1186/s12906-018-2197-6 (PMC5913799; doi:10.1186/s12906-018-2197-6)
Supplement: Supplementary file 4 — Percentage of U251 cell growth inhibition by plant extracts of D. moniliforme (triplicate data). (DOCX 11 kb) [file 12906_2018_2197_MOESM4_ESM.docx]

**Additional file 4**

Percentage of U251 cell growth inhibition by plant extracts of *D. moniliforme* (triplicate data).

| **Concentration of extract in μg/ml** | **DME** | **DMM** |
| --- | --- | --- |
| 800 | 52.13 | 42.55 |
| 800 | 51.77 | 37.30 |
| 800 | 51.00 | 39.00 |
| 400 | 23.94 | 27.82 |
| 400 | 26.33 | 25.67 |
| 400 | 25.25 | 25.00 |
| 200 | 10.54 | 18.71 |
| 200 | 17.78 | 22.22 |
| 200 | 15.67 | 20.33 |
| 100 | 8.91 | 4.18 |
| 100 | 3.61 | 4.82 |
| 100 | 5.67 | 4.55 |
