# Supplementary figures and images for: Antioxidant and cytotoxic activities of Dendrobium moniliforme extracts and the detection of related compounds by GC-MS
Source: BMC Complement Altern Med. 2018 Apr 23;18:134. doi: 10.1186/s12906-018-2197-6 (PMC5913799; doi:10.1186/s12906-018-2197-6)

**Additional file 5**

Chromatogram of *D. moniliforme* methanol extract (DMM).


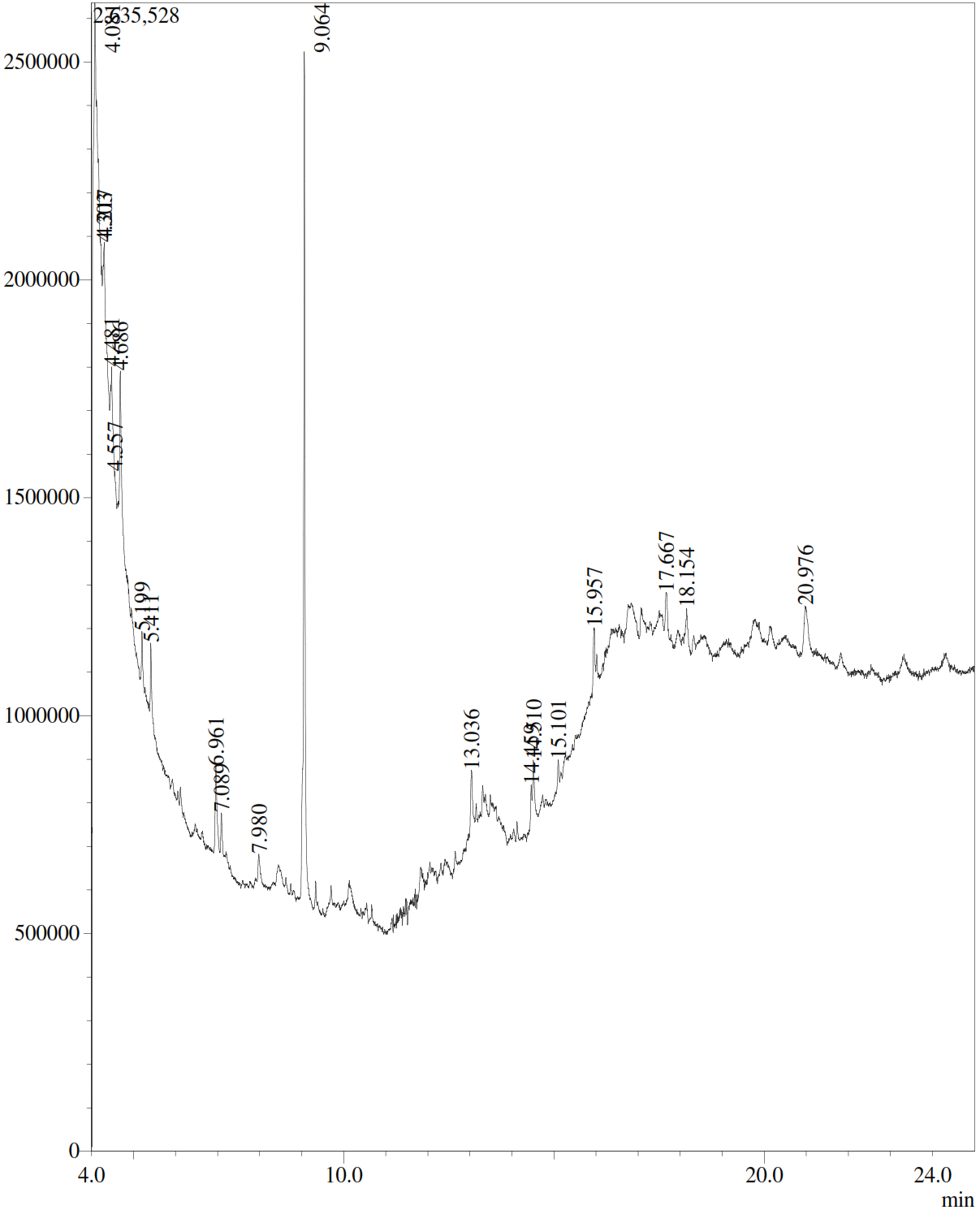

Supplement: Supplementary file 5 — Chromatogram of D. moniliforme methanol extract (DMM). (DOCX 142 kb) [file 12906_2018_2197_MOESM5_ESM.docx]

**Additional file 6**

Chromatogram of *D. moniliforme* ethanol extract (DME).


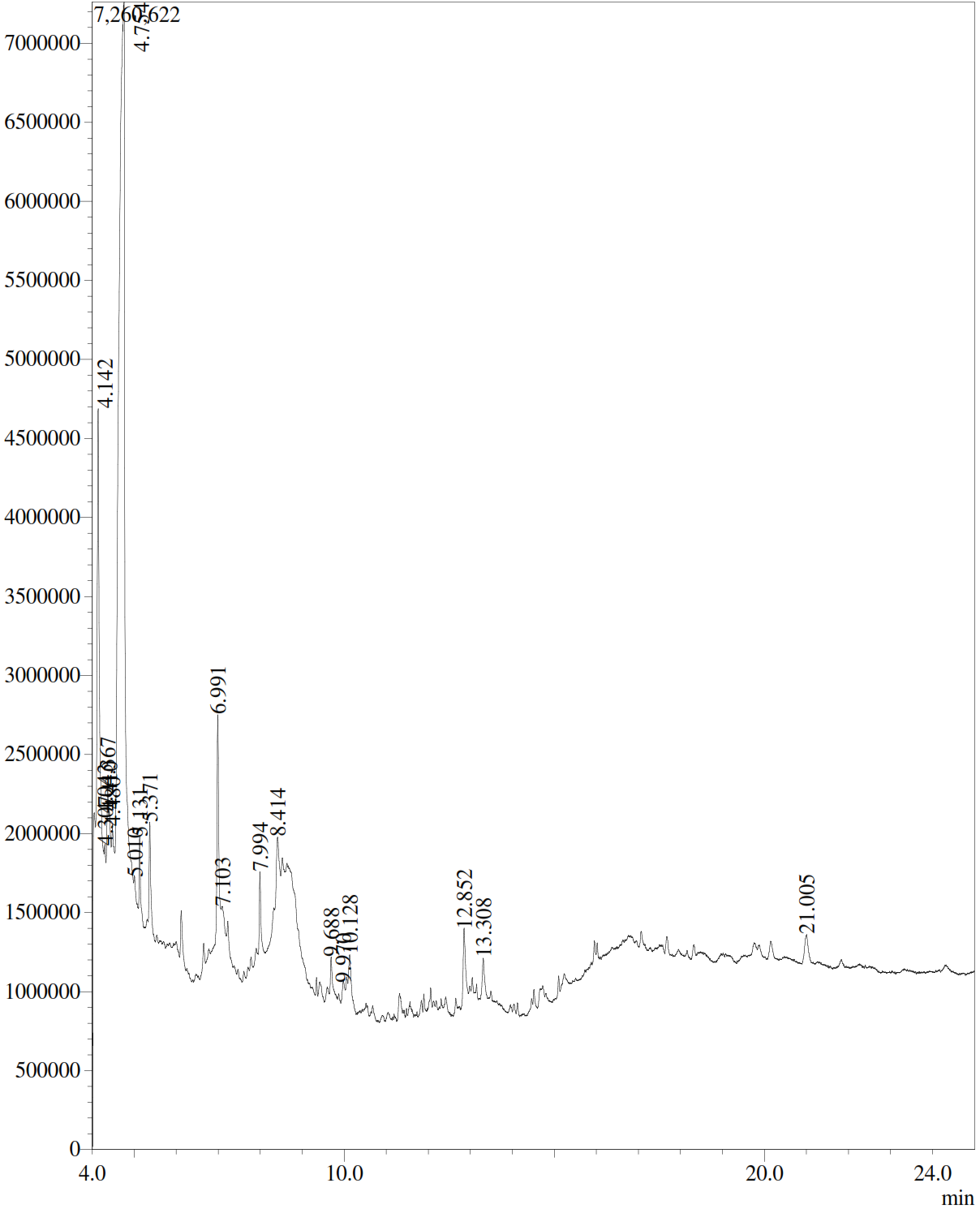

Supplement: Supplementary file 6 — Chromatogram of D. moniliforme ethanol extract (DME). (DOCX 143 kb) [file 12906_2018_2197_MOESM6_ESM.docx]
